# Post-Operative Atrial Fibrillation: Current Treatments and Etiologies for a Persistent Surgical Complication

**DOI:** 10.26502/jsr.10020209

**Published:** 2022-03-28

**Authors:** Leilani A Lopes, Devendra K Agrawal

**Affiliations:** 1 Western University of Health Sciences, College of Osteopathic Medicine of the Pacific - Northwest, Lebanon, OR, USA; 2 Western University of Health Sciences, College of Osteopathic Medicine of the Pacific, Pomona, CA, USA

**Keywords:** Atrial fibrillation, Cardiac surgery, Inflammation, Oxidative damage, Post-operative atrial fibrillation, POAF

## Abstract

Post-operative atrial fibrillation (POAF) is a persistent and serious surgical complication that occur in 20-55% of cardiac surgery cases. POAF may lead to adverse health outcomes such as stroke, thromboembolism, cardiac arrest, and mortality, and may develop long-term. Patients have a 2-fold increase in mortality risk and spend about 3.7 more days in the hospital and $16,000 more in medical costs during their visit. The mechanisms and risk factors of POAF are still poorly understood, yet a strong foundation of how a disease process occurs is needed to provide the most effective treatment. Current mechanisms that are postulated to contribute to POAF include an increase in sympathetic tone, oxidative stress, local and systemic inflammation, a trigger that induces atrial substrate changes, a driver to sustain POAF, and electrolyte disturbances such as hypomagnesemia. While needing more research, current risk factors include age, male sex, history of myocardial infarction or heart failure, hypertension, diabetes, obesity, and COPD. Treatments mostly include prophylaxis of repurposed drugs such as beta-blockers, statins, oral anticoagulants, antiarrhythmics, and Vitamin D and electrolyte supplementation. Autonomic denervation has also been a promising preventative measure for patients undergoing cardiac surgery. This critical review article provides an up-to-date and comprehensive summary of the pathophysiology of POAF, current clinical risk factors and management for POAF and discusses new pathways for further investigation.

## 1. Introduction

In spite of many advances in perioperative, surgical care, and medical innovation there have been complications that continue to persist, such as atrial fibrillation occurring post-operation. Post-operative atrial fibrillation (POAF) is a common, expensive, and potentially fatal complication arising in 20-55% of cardiac surgery cases [1,2]. In about 90% of patients who develop POAF it occurs within the first six days post operation, corresponding with the peak of the postoperative systemic inflammation response [3]. POAF is a predictor of early- and long-term cardiovascular complications such as stroke, thromboembolism, infection, cardiac arrest, and the need for reoperation due to internal bleeding [4,5]. Patients who develop POAF have a two-fold increased risk in all-cause 30-day and 6-month mortality and spend 3.7 more days in the hospital on average than patients who do not develop POAF [6-8]. In the United States the clinical and financial burden for POAF complications persists, with an annual healthcare expenditure over $1 billion and extended hospital stays, presenting a significant issue for patient and their families [3,6,9]. Despite an effort to debunk the reason why patients develop atrial fibrillation (AF) following post-surgical intervention, the etiology and risk factors of POAF are poorly understood. Medical prevention and treatment usually follow an understanding of etiology and considering that the mechanism of pathology is still ambiguous, there are limited successful preventative measures and medical treatments for POAF. This critical review provides a comprehensive and up-to-date summary of the etiologies, risk factors, methods, long-term effects, and financial burdens of POAF and discusses the gaps in our knowledge of the underlying cellular and molecular mechanisms of POAF.

## 2. Methods

In this review, comprehensive literature searches were done on PubMed and Google Scholar from January 2004 through January 2022. Key words included: post-operative atrial fibrillation, cardiothoracic surgery, inflammation, oxidative damage, and atrial fibrillation, POAF treatment. Only the articles in English language were reviewed.

### 2.1 Proposed Etiology of POAF

#### 2.1.1 Trigger and Atrial Substrate Changes:

Post-operative atrial fibrillation typically presents differently from other forms of AF. POAF typically develops within the first 6 days post-operation and then returns to normal sinus rhythm. While the definitive mechanism of POAF has remained elusive, current etiologies that are presumed to contribute to POAF usually require a trigger and a vulnerable atrial substrate change, typically originating from the pulmonary veins and other atrial locations [10,11]. In addition, several biochemical events leading to metabolic derangements in cardiomyocytes affect structural, contractile, and electrophysiological cellular properties in the pathogenesis of AF [12,13].

#### 2.1.2 A Driver to Sustain POAF:

Along with a trigger and a vulnerable atrial substrate, another probable mechanism that contributes to POAF is the presence of a driver that will sustain atrial fibrillation in its vulnerable substrate. Ordered re-entry from re-entrant circuits of short cycle length, a rapidly firing ectopic pacemaker (atrial or junctional), or random re-entry by multiple re-entrant wavelengths may sustain POAF in the presence of a vulnerable substrate [11].

#### 2.1.3: Sympathetic tone, Inflammation and Oxidative Stress:

Activation of the complement system releases pro-inflammatory cytokines such as C-reactive protein (CRP), interleukin-2 (IL-2) interleukin-6 (IL-6) and tumor necrosis factor-α (TNF-α) that follow a close time of occurrence to the onset of POAF [14]. These inflammatory markers result in leukocyte activation and release oxidases and nitric oxide. Eventually, this leads to generation of reactive oxygen species that develop into systemic inflammation [15]. Along with systemic inflammation, pericardial fluid (PCF) volume increases after cardiac surgery and causes local inflammation around the heart. Additionally, the myocardium itself contributes to local inflammation by releasing pro-inflammatory cytokines and has a direct effect on the heart itself. Local inflammation that occurs within the pericardial space results in apoptosis of cardiomyocytes as well as a change in electrical activity, which can cause propagation of arrhythmias and altered action potentials [1]. However, icosapent ethyl (IPE), a marine-derived omega-3 fatty acid, has been shown to be a powerful anti-inflammatory medication, yet patients are at a higher risk for developing atrial fibrillation [16]. If POAF is caused by local or systemic inflammation, why do drugs that decrease the levels and activity of inflammatory markers lead to a significantly increased risk of atrial fibrillation? Along with local and systemic inflammation, an overdrive of sympathetic tone has been postulated to contribute to POAF. Sympathetic drive in the heart results from norepinephrine or epinephrine binding to β1-adrenergic receptors, which is stimulated from the cervical and upper thoracic sympathetic chain ganglia to the deep and superficial cardiac plexus. An increase in adrenergic drive and a decrease in vagal tone could shorten the PR interval and atrial wavelength, increase the inotropic state and preload, and may potentially trigger POAF [17-19]. A mechanism of unrelieved pain, which typically occurs after surgery, involves the mobilization of the sympathetic nervous system. Pain, especially unrelieved pain, could be a trigger for atrial fibrillation. However, in a placebo-controlled clinical trial, Dexmedetomidine for reduction of atrial fibrillation and delirium after cardiac surgery (DECADE), the levels of pain and opioid consumption was unremarkable between patients who developed atrial fibrillation post-operation versus patients who did not [20]. Because of the multifactorial nature of atrial fibrillation and delirium, the findings from this study suggest no association between opioid use and atrial fibrillation and delirium. An increase in global cardiac strain is also driven by the activation of sympathetic tone and the presence of inflammatory markers which are increased by transfusions during cardiac surgery. In a recent study 43% of patients developed POAF and showed increased post-operative fluid balance and M-terminal pro-brain natriuretic peptide (NT-ProBNP). Also, anemia pre-operation led to increased transfusion volumes, and suggests that POAF may be triggered by global cardiac strain [20].

#### 2.1.4 Electrolyte Disturbances:

Hypomagnesemia is a common feature post-surgery and has been shown to be a biomarker for predicting POAF. TRPM7 channels, which play a vital role in converting fibroblasts to myofibroblasts, are involved in fibrogenesis that occurs in POAF. These channels are typically activated by low concentrations of free magnesium [22]. In the Framingham Heart Study, it was shown low magnesium level after cardiac surgery was an indication for the onset of POAF [23,24]. A meta-analysis showed that treating patients prophylactically with magnesium in the post-operative period only led to a statistically significant decrease in POAF [25], suggesting the use of intravenous magnesium as an alternative to prevent POAF. However, careful randomized clinical studies are required to determine the safety and duration of effect.

### 2.2 Post-Operative Atrial Fibrillation Risk Factors

POAF after cardiac surgery is a persistent surgical complication and increased in patients who have comorbidities. The most common risk factors that contribute to POAF are hypertension, age, history of myocardial infarction, obesity, heart failure, male sex, type of surgery, atrial enlargement or block or valvular heart disease [18].

#### 2.2.1 Type of Surgery:

POAF presents a major risk for any cardiac surgery; however, some surgeries are more likely to induce POAF. Specifically, patients who undergo combined surgeries, such as a combined valve and coronary artery bypass graft (CABG) surgeries, are twice as likely to develop POAF, up to 60% [25]. CABG surgeries alone lead to a higher risk for developing POAF, and patients experience an increased risk of other complications from POAF [26,27]. An observational study following patients for 10 years post-CABG showed an increase in stroke, long-term AF, and overall and cardiac mortality [28]. In the Total Arch Repair (TAR), a challenging cardiac surgery, there is a 32.3% incidence of POAF with the highest risk population found with elderly women who underwent a longer operation. Therefore, the type of surgery and length of the surgery are shown to be significantly correlated with the onset of POAF [29].

#### 2.2.2 Pre-existing Cardiac Comorbidities:

An extensive patient history is vital in any cardiac case, especially a surgical case. POAF has many risk factors that include pre-existing cardiac medical conditions or prior episodes such as hypertension, diabetes, chronic obstructive pulmonary disease (COPD), heart failure, and history of myocardial infarction. A meta-analysis showed that these conditions are early predictors of POAF [30,31]. Shown in table 1 are the associated pre-existing cardiac conditions from Yamashita et. al. [30] with their percentage of increased risk to POAF. History of heart failure contributed to the greatest increase in risk for POAF following cardiac surgery. Left atrial enlargement and poor contraction, valvular heart disease and impaired left ventricular function all can contribute to heart failure and aortic stenosis [32,33]. Diabetes had the least increased risk of POAF at 6%, with an odds ratio of 1.06 and (95% CI: 1.00 to 1.13). Dyslipidemia did not show any association with POAF in this meta-analysis [32].

#### 2.2.3 Non-Cardiac Risk Factors:

Increasing age has a strong correlation with the onset of POAF. Literature has discussed that as age advances, the development of an atrial substrate increases which may induce POAF. Age has also been associated with a slowing of conduction rate and increase in fibrosis. Some studies have suggested that male sex is an independent risk factor for developing POAF, however there is conflict in the literature. A comparative study showed that men had an increased postoperative peak lactate and a higher incidence of death after a four-year follow up [34]. Caucasian patients who underwent CABG also have been shown to have a higher incidence of POAF than non-caucasian patients. However, studies that compare race and POAF use a significantly lower patient population of non-white to white patients [35].

#### 2.2.4 Modifiable Risk Factors:

Obesity and high BMI is a persistent risk factor in many cardiac pathologies, including POAF. Obesity and high BMI put patients at risk for hypertension and diabetes, and therefore may be a secondary risk factor for developing POAF. A systematic review and meta-analysis showed that of the patients that went cardiac surgery, patients with a high BMI or obesity showed to have a significantly increased risk of developing POAF than patients without these risk factors (p = 0.006) [36].

#### 2.2.5 CHADS2 and CHAD2DS2-VASc score:

The CHADS2 (Congestive heart failure, Hypertension, Age (> 65 = 1 point, > 75 = 2 points), Diabetes, previous Stroke/transient ischemic attack (2 points)) and CHAD2DS2-VASc (congestive heart failure, hypertension, age ≥75 (doubled), diabetes, stroke (doubled), vascular disease, age 65 to 74 and sex category (female)) scores are independent predictors of POAF with a strong selectivity and sensitivity for POAF [37]. These scores determine a patient’s stroke risk by combining cardiovascular and non-cardiovascular factors and are useful for predicting POAF. Other scores such as HATCH (hypertension <1 point>, age >75 years <1 point>, stroke or transient ischemic attack <2 points>, chronic obstructive pulmonary disease <1 point>, and heart failure <2 points>) score have been used. However, score using the combination of variables with higher predictive value included in the POAF (COM-AF) has been reported to be more useful for predicting POAF development [11].

### 2.3 Methods for Reducing POAF Incidence

#### 2.3.1 Beta-Blockers:

**Figure 1:**
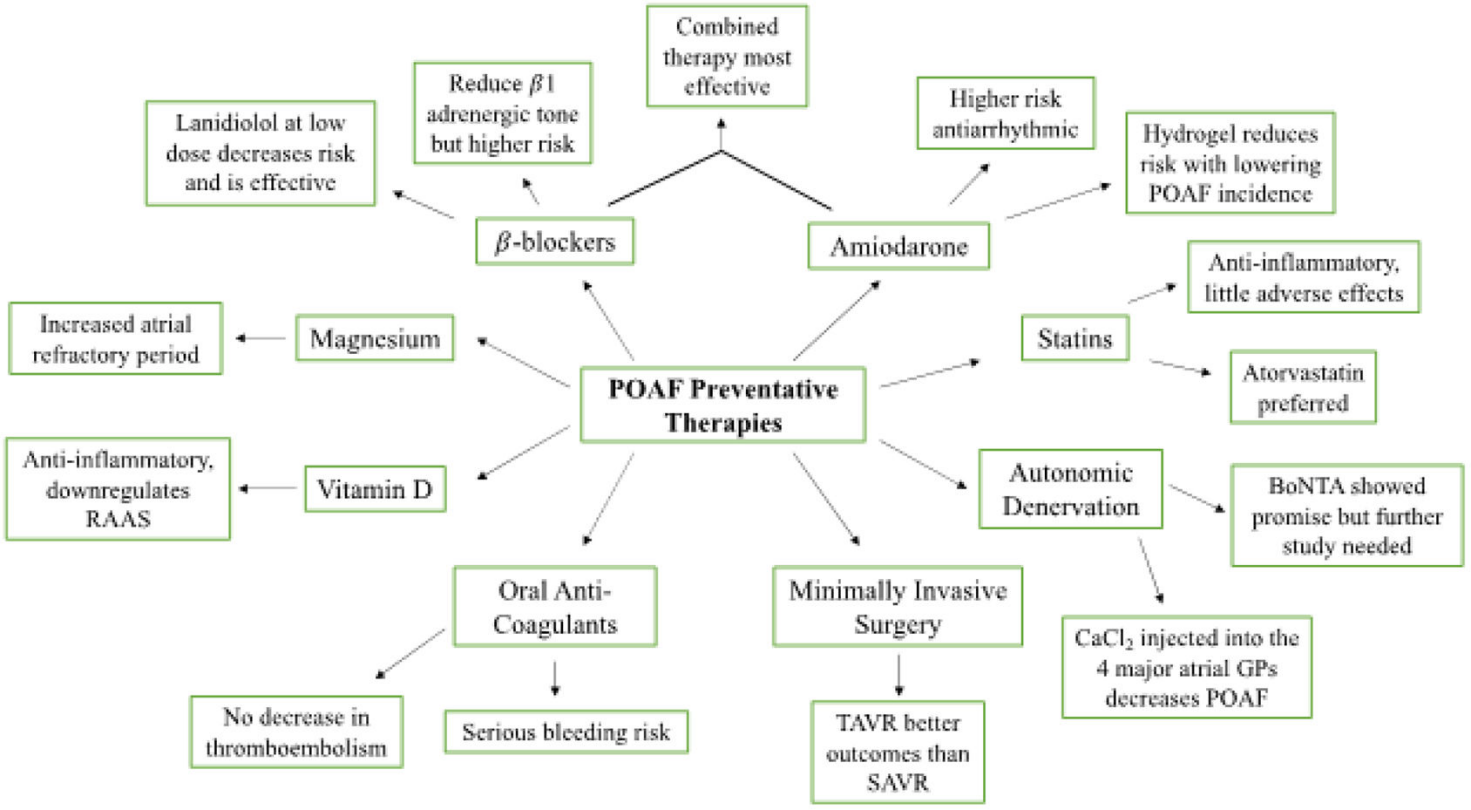
An overview of the proposed preventative therapies for POAF. Pharmacological intervention includes β-Blockers, amiodarone, statins, and oral anti-coagulants. Surgical intervention includes autonomic denervation and minimally invasive surgery. Vitamin D and magnesium supplementation were also proposed as a preventative therapy for reducing the incidence of POAF.

Beta-1 receptor blockers or antagonists inhibit β1-adrenergic receptors responsible for sympathetic stimulation in the myocardium. One of the etiologies of POAF is by an increase in sympathetic tone, which causes an increase in preload and inotropy, thereby increasing the end diastolic volume (EDV) or decreasing the end systolic volume (ESV), respectfully. Either or both phenomena increase the stroke volume (SV) and cardiac output (CO). Beta-blockers inhibit these receptors, thereby decreasing the SV and CO and decreasing the heart rate, downregulating sympathetic tone [38], β-Blockers prophylaxis before cardiac surgery has been shown to be one of the most effective treatments for POAF so far [15]. In a study using β-Blockers in combination with Amiodarone, there was a 13.75% decrease in the onset of POAF with an onset frequency of 17.50% compared to β-Blockers alone at 31.25% as shown in figure 1. β-Blockers in combination with Rosuvastatin, which has proved to be less effective, had a higher percentage of patients who developed POAF at 33.75%. While the sample size was small with a total of 240 patients, this may provide insight into personalized therapies for POAF. Another clinical trial should be conducted with Atorvastatin and β-Blockers to see how this treatment compares to β-Blockers combined with Amiodarone [39]. Although effective against POAF and granted a Class I indication, β-Blockers can lead to a variety of adverse outcomes, most commonly bradycardia and hypotension. While β-Blockers are an effective drug against POAF, especially when coupled with Amiodarone, the search continues for the perfect treatment due to the lack of knowledge about the etiology of POAF and some of the adverse effects of β-Blockers. Lanidiolol hydrochloride is a low-dose β-selective blocker that has been shown to give a negative ionotropic effect without the adverse outcomes of β-blockers at a low dose. While debated in literature, The PELTA (Preventive effect of low-dose Lanidiolol on postoperative atrial fibrillation) study showed landiolol has a preventative effect against POAF at low doses in patients who undergo CABG, valvular and aortic surgery [40].

#### 2.3.2 Amiodarone:

Amiodarone belongs to the class of antiarrhythmics and is a peripheral and coronary vasodilator [41]. It is granted Class IIA indication by both American and European guidelines and is one of the most effective treatments for POAF [42]. However, amiodarone presents with serious cardiac and extracardiac adverse risks, such as bradycardia, increased liver enzymes, hyperthyroidism, thyrotoxicosis, and interstitial pneumonitis. Accumulation of the drug may also lead to longer lasting side effects after discontinuing the drug [7]. Local epicardial application of amiodarone hydrogel has been shown to reduce the adverse outcomes of amiodarone and have a decrease in POAF incidence from 22-37% to 3.3-8% [7,42]. While these studies were small in sample size (~ 60), they show promise for an effective therapeutic for POAF.

#### 2.3.3 Statin Therapy:

Statins, independent from their lipid-lowering properties, also have anti-inflammatory effects. Clinical trials have shown that statins significantly reduce C-reactive protein (CRP), tumor necrosis factor-α (TNF-α), Interleukin- 6 (IL-6) and Interleukin 1 (IL-1) versus placebo. Recently in literature, Atorvastatin and Rosuvastatin have been under study for reducing the incidence of POAF. Benefits are that they have less side-effects than other pharmaceuticals that are being used to mitigate POAF. Some studies have shown that Rosuvastatin given perioperatively had no effect on POAF incidence compared to placebo. However, multiple meta-analyses have shown that statins are effective at preventing POAF in patients undergoing CABG, with Atorvastatin showing effectiveness instead of Rosuvastatin [43,44].

#### 2.3.4 Oral Anticoagulants:

POAF onset after CABG surgery has been associated with a higher risk thromboembolism, and recent studies have postulated using oral anti-coagulants (OAC) as a post-operative therapeutic to reduce the risk of thromboembolism once POAF develops [12,45]. Multiple trials, including the SWEDEHEART Registry clinical trial, showed that OAC did not decrease the incidence of thromboembolism in POAF patients and increased the rate of major bleeding [46-48]. Early anticoagulation therapy is a current Class IIA recommendation from international guidelines as preventative treatment for thromboembolism; however, more studies need to be done to determine if the benefits of OAC outweigh the risk of major bleeding or stroke.

#### 2.3.5 Vitamin D Supplementation:

Vitamin D is a critical sterol in calcium metabolism and mobilization.Traditionally believed to be limited to bone tissue, Vitamin D receptors (VDRs) have found throughout the body, including cardiomyocytes [49,50]. It has been shown that Vitamin D is invaluable for cardiovascular health, and since most of the United States is deficient in Vitamin D, it has become a recent method for helping prevent cardiomyopathies and infection [51]. Vitamin D has powerful anti-inflammatory abilities such as prostaglandin and cyclooxygenase pathway inhibition, renin-angiotensin-aldosterone system (RAAS) downregulation and anti-inflammatory cytokine upregulation [52]. RAAS activation increases and electrically and structurally remodels the atria, contributing to POAF. In multiple studies, supplementation with Vitamin D lowered the prevalence of POAF via RAAS inhibition [13,52].

#### 2.3.6 Magnesium Supplementation:

Supplementation with Mg significantly increases the refractory period of atrial repolarization. The proposed mechanism for hypomagnesium contributing to POAF is due to dilution from transfusion during surgery [47]. There has been debate in the literature about magnesium supplementation for treatment of POAF, and a meta-analysis showed that post-operative magnesium supplementation was the only time frame that significantly reduced the risk of POAF [24].

#### 2.3.7 Autonomic Denervation:

In the epicardial fat pads lie the ganglionated plexi (GPs) that act as integrated centers for autonomic innervation and act on the sinoatrial and atrioventricular node. Since it had been shown that simultaneous or successive stimulation of both the parasympathetic and sympathetic nervous systems in cardiac tissue induce POAF, it was hypothesized whether denervation of GPs adjacent to the pulmonary veins, such as in AF ablation, would decrease the incidence of POAF. A study of 200 patients undergoing CABG had CaCl2 injected into the 4 major atrial GPs and reduced the risk of POAF from 36% to 15%, yet length of hospitalization did not change between the two groups. Heart rate variability data showed that sympathetic and parasympathetic balance was undisturbed by the injection. Onabotulinum toxin A (botulinum toxin type A [BoNTA]) was also tested to denervate GPs in epicardial fat pads. A randomized, placebo-controlled, double-blind clinical trial containing 145 patients showed a decrease incidence of POAF compared to placebo from 47.8% down to 36.5%. Though shown in this trial not to be statistically significant, BoNTA does seem to have promise in reducing POAF, especially due to the injection having little adverse effect (Waldron et. al. 2019). However, other trials have shown that POAF is decreased by almost half (Romanov et. al. 2019). Another study should be conducted with a higher patient population in contrast with CaCl2 on POAF onset post-cardiac surgery.

#### 2.3.8 Opting for Minimally Invasive Cardiac Surgery:

Minimally invasive surgery has been postulated to decrease incidence of POAF. CABG surgery has the highest incidence for developing POAF and other long-term effects and methods to reduce the incidence of POAF in CABG patients has been the focus for many clinical trials. A follow-up study comparing minimally invasive direct coronary artery bypass (MIDCAB) showed no correlation to POAF [53]. Transcatheter Aortic Valve Replacement (TAVR) is a method that is increasing in popularity over Surgical Aortic Valve Replacement (SAVR), due to it being less invasive [1,54]. In the PARTNER3 trial, TAVR had a lower incidence of early POAF that patients who underwent SAVR. The study also showed that later onset POAF had a higher incidence of adverse outcomes despite either treatment method [55]. Other studies supported this finding, suggesting that the less invasive TAVR led to a decreased risk of POAF than traditional SAVR procedures [56].

### 2.4 The Financial and Clinical Burdens of Patients with POAF

While the definitive mechanism and solution for POAF remains elusive, the complication presents a heavy burden for patients, hospitals, and families. One study reported that patients that develop POAF after CABG are twice as likely to die (p < 0.01) [3,4]. Not only does POAF contribute to a higher mortality, but it also causes a large financial burden for patients and their families. A randomized control trial showed that the 1-year cost difference for patients that developed POAF was +$15,593 USD due to a longer hospital stay and complications arising from POAF [4,57]. Therefore, attempts to reduce the incidence of POAF by understanding the mechanism of the complication or finding a consistent preventative measure for reducing POAF could help decrease hospital resource utilization and patient expenses after cardiac surgery [58-65].

## 3 Conclusion

POAF is a common and expensive complication that occurs in 20-55% of cardiac surgery patients and has increased risk for stroke, thromboembolism, and mortality. Risk factors and etiologies for POAF are poorly understood. However, risk factors that have shown to be significant include type of surgery, age, diabetes, hypertension, history of heart failure, COPD, obesity, male sex, and myocardial infarction. Proposed mechanisms for the development of POAF include atrial substrate changes when exposed to a trigger and/or a driver to sustain POAF, sympathetic stimulation, local and systemic inflammation, oxidative stress, and electrolyte disturbances. Current treatments are typically via drug repositioning such as statins, beta-blockers, antiarrhythmics, oral anticoagulants and restoration of electrolytes. Some research has also been done on autonomic denervation, yet more studies are needed to determine the efficacy and long-term effects of this treatment. POAF also provides a large burden by increasing the cost of a patient’s hospital stay by about $15,593 on average and increasing the use of hospital resources. Due to the prevalence of POAF after cardiac surgery, it is vital that the mechanism of POAF is elucidated so that patients can receive preventative treatment.

## Figures and Tables

**Table 1:** Pre-existing cardiac conditions that predispose patients to POAF. Interpreted from Yamashita et al. (2019).

	Risk Increase for POAF	Odds Ratio
Hypertension	29%	1.29 (95% CI: 1.12 to 1.49)
Diabetes	6%	1.06 (95% CI: 1.00 to 1.13)
COPD	36%	1.36 (95% CI: 1.13 to 1.64)
Heart Failure	56%	1.56 (95% CI: 1.31 to 1.86)
Myocardial Infarction	18%	1.18 (95% CI: 1.05 to 1.34)

## Abbreviations

AF: Atrial fibrillation
CABG: Coronary artery bypass grafting
CO: Cardiac output
EDV: End diastolic volume
ESV: End systolic volume
OACs: Oral anticoagulants
PCF: pericardial fluid
POAF: Post-operative atrial fibrillation
RAAS: renin-angiotensin-aldosterone system
SAVR: Surgical aortic valve replacement
SV: Stroke volume
TAR: Total arch repair
TAVR: Transcatheter aortic valve replacement
VDRs: Vitamin D receptors
